# Automatic software correction of residual aberrations in reconstructed HRTEM exit waves of crystalline samples

**DOI:** 10.1186/s40679-016-0030-1

**Published:** 2016-11-30

**Authors:** Colin Ophus, Haider I Rasool, Martin Linck, Alex Zettl, Jim Ciston

**Affiliations:** 1 National Center for Electron Microscopy, Molecular Foundry, Lawrence Berkeley National Laboratory, 1 Cyclotron Road, Berkeley, USA; 2 Department of Physics, University of California Berkeley, 366 LeConte Hall, Berkeley, MC 7300 USA; 3 Materials Science Division, Lawrence Berkeley National Laboratory, 1 Cyclotron Road, Berkeley, USA; 4 Corrected Electron Optical Systems GmbH, Englerstrasse 28, 69126 Heidelberg, Germany

**Keywords:** Atomic resolution HRTEM, Aberration correction, Inline holography, Off-axis holography, Wavefront sensing

## Abstract

We develop an automatic and objective method to measure and correct residual aberrations in atomic-resolution HRTEM complex exit waves for crystalline samples aligned along a low-index zone axis. Our method uses the approximate rotational point symmetry of a column of atoms or single atom to iteratively calculate a best-fit numerical phase plate for this symmetry condition, and does not require information about the sample thickness or precise structure. We apply our method to two experimental focal series reconstructions, imaging a β-Si_3_N_4_ wedge with O and N doping, and a single-layer graphene grain boundary. We use peak and lattice fitting to evaluate the precision of the corrected exit waves. We also apply our method to the exit wave of a Si wedge retrieved by off-axis electron holography. In all cases, the software correction of the residual aberration function improves the accuracy of the measured exit waves.

## Background

Hardware aberration correction for electron beams in transmission electron microscopy (TEM) is now widespread, substantially improving the interpretable resolution in TEM micrographs [1–4]. This technology is enabled by the combination of two factors; the ability to accurately measure optical aberrations in the electron beam, and a system of multipole lenses that can compensate for these measured aberrations. Many authors have studied the problem of direct aberration measurement, and most solutions involve capturing a Zemlin tableau [5–8]. This method requires a thin, amorphous object that can approximate an ideal weak-phase object. Many samples of interest however are partially or fully crystalline. Thus, aberrations must be measured and corrected on an amorphous sample region before micrographs can be recorded on the region of interest. During this delay, the aberrations may drift due to electronic instabilities in the microscope [9], and this factor coupled with imperfect hardware correction can lead to residual aberrations in the resulting electron plane wave measurements.

One possible solution is to reconstruct the complex electron wavefunction via inline holography, by taking a defocus series and employing an exit wave reconstruction (EWR) algorithm such as Gerchberg-Saxton or the Transport of Intensity Equation [10–16]. Alternatively, an exit wave can be reconstructed by interferometric methods, i.e. off-axis electron holography [17, 18]. We can then estimate the residual aberrations and apply a numerical phase plate to the reconstructed complex wavefunction to produce aberration-free images [19]. These numerical corrections fall into two categories; manual correction, where the operator attempts to determine the aberrations present by trial and error, and automatic correction where the aberrations are directly measured in some manner. While the theory of aberration determination from a thin, amorphous sample is well-understood (and used to calibrate the hardware corrector on a modern TEM) [20–22], purely crystalline samples are much more difficult to correct due to the sparsity of diffraction space information [23]. If the sample is a low-index zone axis image of a crystal, there is no simple Fourier space technique to measure residual aberrations for a sample of unknown thickness or composition. Some authors have proposed using entropy methods [24] or measuring atomic column asymmetry within Fourier space [25] to measure residual aberrations. However, the first method requires well-separated atomic columns and the second can have difficulty measuring multiple simultaneous aberrations. We also note that some authors have used converged scanning transmission electron microscopy (STEM) probes to directly evaluate the aberration coefficients from crystalline samples [26–28], but these methods are not directly applicable to plane wave TEM measurements.

In this study, we propose a new method to measure aberrations from TEM images of crystalline samples containing on-axis atomic columns or single atoms. We use these measurements of residual aberrations to iteratively correct the complex exit wave until convergence is reached. Our method requires only a rough guess of the projected crystal structure and a regular (undefected) crystalline region in the image field of view. We test this method on three experimental datasets, focal series reconstructions of a β-Si_3_N_4_ wedge with O and N doping and a single-layer graphene grain boundary, and an off-axis hologram measurement of a Si wedge.

## Theory

### Calculating images with radial point symmetry

**Fig. 1:**
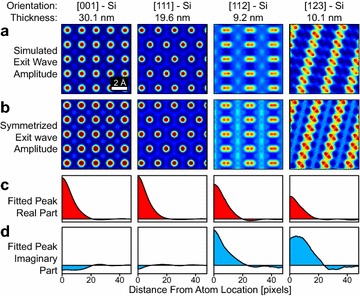
**a** Simulated exit waves of Si at different thicknesses and zone axes. **b** Symmetrized exit waves from **a**. **c**, **d** Real and imaginary parts of fitted atomic shape functions

HRTEM images of thin, crystalline samples oriented along low-index zone axes usually have a high degree of radial point symmetry, around each atomic (or atomic column) coordinate. When multiple peaks are close together, interference between adjacent columns can create amplitude or phase images that appear to break the radial symmetry. However this symmetry breaking is often due to constructive and destructive interference of the underlying complex wave, and the overall exit wave can still be well-described as a sum of isolated, radially-symmetric complex atomic shape functions. To demonstrate this, we have simulated several examples of exit waves of a silicon sample using the multislice method [29], the amplitudes of which are plotted in Fig. 1a.

The first two simulations in Fig. 1a, the [001] and [111] zone axes, have equally spaced atomic columns which show local radial symmetry around each peak. The third and fourth simulations in Fig. 1a contain Si dumbbells and appear to have broken radial symmetry at much shorter distances. These images however can be well-described by a sum of identical, radially-symmetric atomic peak shape functions, shown in Fig. 1b–d.

A point-symmetrized image can be calculated using a few simple steps. First, the atomic coordinates must be estimated (from a known structure) or fitted to the image. Each exit wave pixel value $\psi (x,y)$ is equal to

1 $$\begin{aligned} \psi (x,y)=A_0+\sum _{j=1}^J\sum _{k=1}^{K_J}s_j\left[ \sqrt{(x-x_k^j)^2 + (y-y_k^j)^2} \right] , \end{aligned}$$

where $A_0$ is a constant carrier wave value, there are *J* atom types included, $\mathbf {s}_j(|(x,y)|)$ is the complex atomic shape function for each atom type *J*, and there are $K_J$ atoms of type *J*, located at coordinates $(x_k^j,y_k^j)$.

Next, we calculate an atomic distance matrix $\mathbf {A}$ which relates all image pixels to their distances to all nearby atomic coordinates. Each row of this matrix corresponds to a different image pixel (*x*, *y*), while the columns represent all possible (rounded) distances to all nearby atomic sites, divided up into different atomic species. This matrix is moderately sparse, where the only non-zero values are ones in the first column (corresponding to $A_0$) and ones at the rounded distances of all atoms within some cutoff radius. This formalism allows us to solve for discretized atomic shape function(s) $\mathbf {s}_j$ using the set of linear equations given by

2 $$\begin{aligned} \mathbf {A} \begin{bmatrix} A_0 \\ s_1 \\ \vdots \\ s_J \end{bmatrix} = \psi (x,y) , \end{aligned}$$

which can be solved using typical regression methods. This symmetrization method has been applied in the examples shown in Fig. 1b, where the fitted atomic shape functions are given in Fig. 1c, d. In all cases, the symmetrized exit wave is in perfect or good agreement with the original exit waves shown in Fig. 1a. This simple method for calculating point-symmetrized exit waves forms the basis of the aberration correction algorithm presented here. Note that while we have chosen to solve the peak shape functions in real space, it is also possible to deconvolve a point spread function in Fourier space, similar to the method described by van den Broek et al. [30]. The real-space method simplifies handling of the boundary conditions (by simply not including pixels that are not surrounded by enough atomic coordinates) and can easily handle multiple atom types. Finally we note that a constant value (setting all non-zero values equal to ones) does not need to be assumed for all atomic sites; instead a complex value at the peak coordinate location can be directly measured from the exit wave, or refined by least squares. This step improves accuracy if the reference region used for solving the residual aberrations has non-constant thickness.

### Coherent wave aberrations

A complex exit wave $\psi (x,y)$ measured with off-axis holography or reconstructed from inline holography that contains residual aberrations described by the Fourier-space aberration function $\chi (q_x,q_y)$ is related to the aberration-free exit wave $\psi _0(x,y)$ by the expression [29]

3 $$\begin{aligned} \Psi (q_x,q_y) = \Psi _0(q_x,q_y) \exp \left[ -i \chi (q_x,q_y) \right] \end{aligned}$$

where $\Psi (q_x,q_y)$ and $\Psi _0(q_x,q_y)$ are the 2D Fourier transforms of $\psi (x,y)$ and $\psi _0(x,y)$ respectively. The vector (*x*, *y*) and $(q_x,q_y)$ represent the real space and Fourier space coordinate systems respectively. The aberration function used here is the basis function

4 $$\begin{aligned} \chi (q_x,q_y)= & {} \sum _{m=0} \sum _{n=0} \left[ \lambda ^2 ({q_x}^2 + {q_y}^2) \right] ^{m+n/2} \nonumber \\&\cdot \left\{ C_{m,n}^x \cos \left[ n \cdot \mathrm {atan2} \left( q_y,q_x \right) \right] \right. \nonumber \\&\left. + \,C_{m,n}^y \sin \left[ n \cdot \mathrm {atan2}\left( q_y, q_x) \right) \right] \right\} , \end{aligned}$$

where $(C_{m,n}^x,C_{m,n}^y)$ are the coefficients of the two orthogonal aberrations of order (*m*, *n*) in units of radians, and $\mathrm {atan2}(q_y, q_x)$ is the arctangent function which returns the correct sign in all quadrants (all combinations of signs of $q_x$ and $q_y$). The radial magnitude of each aberration scales with $|q|^{2m + n}$ and the rotation symmetry is given by *n*. Note that when $n=0$, the aberration is radially symmetric (e.g. constant value, defocus, spherical aberration) and no $C_{m,n}^y$ term is necessary. Various authors use different conventions for dimensioning the coefficients $(C_{m,n}^x,C_{m,n}^y)$ [7, 19, 31]. We also note that this function describes only coherent wave aberrations that are constant over the field of view (aplanatic).

### Estimating residual aberration coefficients

**Fig. 2:**
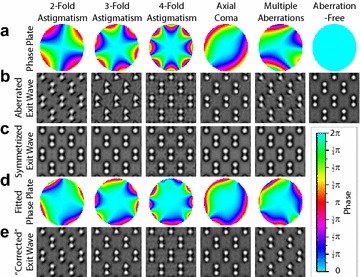
**a** Phase plates for synthetic aberrations applied to simulated Si [011] exit waves, giving **b** amplitudes images. **c** Symmetrized waves corresponding to **b**. **d** Fitted phase plate for aberrations up to 6th order. **e** Exit wave where phase plate in **d** is applied to images in **b**

We now show how symmetrized exit waves can be used to estimate aberrations in images of crystalline samples. As an example, we have simulated exit waves with synthetic aberrations in Fig. 2a, b, for a 19.8 nm thick [011]-Si sample. In all cases except for the aberration-free image, applying an aberration phase plate causes distortions in the atomic images.

Next, a symmetrized image is calculated from the aberrated wave and the approximate peak positions, shown in Fig. 2c. The resulting images appear to be approximately aberration free due to the radial symmetry imposed by constructing an exit wave from radially-symmetric point atomic shape functions, and can be used to estimate the aberration function $\chi (q_x,q_y)$. To generate this estimate, we calculate the windowed Fourier transforms of both the aberrated and symmetrized waves. A window function is used to prevent boundary errors. Next, we measure the difference in phase between the two FFTs and use weighted least squares to fit the aberration coefficients. The weighting function is set to the magnitude of the original exit wave Fourier transform. This ensures that the strongest Bragg components dominate the aberration function fit.

Figure 2d shows the fitted aberration function, including all aberrations up to 6th order. The fits are a good, but not perfect, match to the real aberration functions in Fig. 2a. Applying the fitted aberration functions to the aberrated images produces the images plotted in Fig. 2e. Similar to the fitted aberration function, these images are improved but not yet free of aberrations. This estimation method for the aberration function can be applied iteratively to produce an accurate measurement of the residual aberration functions.

### Iterative algorithm for estimating residual aberrations

**Fig. 3:**
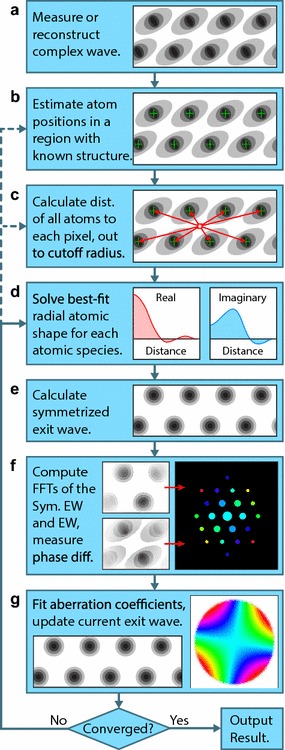
Flow chart for the algorithm proposed in this work, labeled by steps **a**–**g**. All steps must be performed at during the first iteraiton, while additional iterations can begin at steps **b**, **c** or **d**

Our proposed algorithm for correcting residual aberrations in complex exit waves of crystalline samples is diagrammed in Fig. 3. We start with a reference region in the exit wave $\psi (x,y)$. This region should be roughly constant thickness and contain as few lattice defects as possible. Increasing the area of the reference region will improve the accuracy of the fitted aberrations, at the cost of increased computation time. From this reference region, we generate a list of atomic coordinates, and if multiple types of atoms are present, the corresponding site identities.

Next we calculate the distance matrix $\mathbf {A}$ between all pixels in the reference region and the atomic coordinates. This procedure is shown geometrically for a single pixel in Fig. 3c. We then use linear regression to solve for the complex atomic shape function for all species present. The distance matrix $\mathbf {A}$, carrier wave value $A_0$, and the shape functions $\mathbf {s}_1 \ldots \mathbf {s}_J$ are then used to calculate a symmetrized exit wave.

Subsequently, we compute a windowed Fourier transform of the current guess for the aberration-free exit wave (in the first iteration the measured exit wave is used) and the symmetrized wave. We measure the phase difference of these Fourier transforms, shown in Fig. 3f. We use weighted least squares to fit the aberration coefficients, where the Fourier transform amplitude of the exit wave is used as the weighting function. These aberration function coefficients are added to the current values from the previous iteration (originally initialized to zero). This fitted aberration function is then applied to the original exit wave as in Fig. 3g, generating an updated guess for the aberration-free exit wave. If the corrected exit wave update is below a user-defined threshold, we assume the algorithm is converged and output the result. If not, we perform additional iterations.

The algorithm described in Fig. 3 has three possible re-entry points for additional iterations, shown by the dashed lines. If we assume the atomic positions are accurate, we do not need to update them or recalculate the distance matrix $\mathbf {A}$. Since this is the most time-consuming step of the algorithm, skipping it for additional iterations saves most of the calculation time. Alternatively, the atomic positions can be updated by peak fitting or a correlation method, starting the next iteration at the step in Fig. 3b. If the atomic positions are accurate enough, there is one other possible update at the start of each iteration. Each atomic site can be updated with a complex scaling coefficient to approximate slight thickness changes in the reference region. Both of these alternative update steps require updating the distance matrix $\mathbf {A}$, step Fig. 3c.

### Limitations of the method

The algorithm for measuring and correcting residual wave aberrations described above requires a relatively flat, defect-free region within a portion of the full field-of-view. A small reference region will degrade the accuracy of the measured aberration function. In the experimental results shown below, the size of the reference region was $\approx$50 unit cells for the Si_3_N_4_ sample, $\approx$1000 unit cells for the graphene sample, and $\approx$150 unit cells for the silicon wedge. The accuracy of the residual aberration function also depends on the signal to noise and accuracy of the exit wave reconstruction or measurement. If the crystalline region of the sample contains random variation of the exit wave due to an amorphous layer on the surface, or systematic variations due to surface reconstruction, the resulting aberration function may contain small errors. This issue can be minimized by using as large a reference region as possible, and with good sample preparation methods.

Another possible source of error is sample mis-tilt. Completely eliminating sample tilt is virtually impossible, and small amounts of sample tilt can mimic some residual aberration functions, in particular axial coma. Similarly, if the sample thickness changes linearly over the reference region, our method may fit a small amount of erroneous axial coma under some circumstances. However, because both of these effects heavily sample-dependent, it is impossible to assign firm numbers to the possible degree or error. In general we recommend using complementary measurements to verify results, such as measurement of mean atomic coordinates or unit cell dimensions or angles from x-ray diffraction.

## Methods

### Simulations

Multislice simulations were performed using Matlab code following the methods of Kirkland [29]. Unless otherwise noted, all simulations were performed at 300 kV, a pixel size of 0.05 $\mathrm {\AA }$ and 32 frozen phonon configurations. An information limit of 1.5 $\mathrm {\AA }^{-1}$ was enforced by applying an 8th order Butterworth filter to the exit waves in Fourier space. The exit waves were not further defocused after propagation through the sample, approximating a white atom contrast condition for all amplitude images.

### Experiments

The Si_3_N_4_ sample was flat polished on one side using an Allied MultiPrep system, then mirror polished with 0.1 μm diamond paper. The second side was dimpled and finished with a 1.0 μm diamond slurry to a thickness of about 20 μm. The sample was then ion milled on a Gatan PIPS at 0 °C using 5 kV Ar ions at an angle of 5° for 3 h, then at 1 kV for 30 min, followed by 0.5 kV for 5 min. This latter sample was not carbon coated and was found to be stable under the beam operated at 300 kV. Focal series of this sample were recorded at 300 kV in the TEAM 0.5, an FEI TITAN-class microsope [3]. The corrector was tuned for bright atom contrast (C_3_ = −6 μm, C_5_ = 2.5 mm) and the monochromater was excited to provide an energy spread <0.15 eV at full width half maximum. The focal series were acquired with a step size of 1.72 nm ranging from −34.4 nm underfocus to 34.4 nm overfocus, recorded on a Gatan Ultrascan 1000.

The graphene sample was grown at 1035 °C by chemical vapor deposition onto a polycrystalline copper substrate. The substrate was held at 150 mTorr hydrogen for 1.5 h before 400 mTorr methane was flowed over it for 15 min to grow single layer graphene [32]. This sample was imaged in the TEAM 0.5 microscope using mochromated, spherical aberration-corrected 80 kV imaging with the monochromater excited to provide an energy spread <0.15 eV at full width half maximum. A focal series of 5 images with a step size of 1.2 nm was recorded on a Gatan OneView detector.

An off-axis hologram of a silicon wedge was recorded in the Cc-Cs-corrected TEAM I microscope operated at 80 kV accelerating voltage using an exposure time of 8 s on a Gatan Ultrascan 1000. The [011]-silicon sample was laser cut from a 3 mm disc down to as 1 mm to fit the TEAM stage geometry [33]. For hologram acquisition, the corrector was tuned to correct all aberrations, up to and including 3rd order, below the measurement accuracy of the aberrations. The exit wave was reconstructed from the hologram using simple numerical Fourier optics [17].

### Focal series reconstruction and data analysis

All focal series reconstructions and data processing except for the off-axis holographic reconstruction were performed using Matlab code. Focal series reconstructions were performed using the Gerchberg–Saxton algorithm [10] where the implementation for HRTEM is described fully in [11, 12]. During reconstruction sub-pixel image alignments were applied using the discrete Fourier transform method given in [34].

To quantify the atomic column positions in a complex image, we used nonlinear least squares to fit the peak positions using a two-dimensional elliptic Gaussian function for both the real and imaginary components. This peak function $\beta (x, y)$ defined as

5 $$\begin{aligned} \beta (x,y)=\, & {} b_1 \exp \left[ - b_2 \Delta x^2 - 2 b_3 \Delta x \Delta y - b_4 \Delta y^2 \right] \nonumber \\&+ i b_5 \exp \left[ - b_6 \Delta x^2 - 2 b_7 \Delta x \Delta y - b_8 \Delta y^2 \right] \nonumber \\&+ b_9 + i b_{10}, \end{aligned}$$

where $b_1$ through $b_{10}$ are the real fitting coefficients and $\Delta x = x - x_0$ and $\Delta y = y - y_0$ are the distances from the peak center $(x_0,y_0)$. For the Si-N dumbbells, two complex elliptic Gaussian functions were fitted to both peaks simultaneously.

## Results and discussion

### Exit wave reconstruction of Si$_3$N$_4$

The first sample analyzed is a SiAlON wedge sample (isostructural to $\upbeta$-Si$_3$Al$_4$ with Al and O doping the Si and N sites to give the composition Si$_{5.6}$Al$_{0.4}$O$_{0.4}$N$_{7.6}$), recorded at 300 kV along the [0001] direction. Density functional theory [35] and neutron-scattering studies [36] predict that O might preferentially dope the 2a sites with a nearby Al balancing the extra charge, causing a 21 pm shift in one of three directions [37]. X-ray diffraction by contrast shows no site preference for Al or O [38]. We therefore wish to measure the column positions with as high a precision as possible to evaluate the dopant-ordering hypothesis and its potential local variation at the nanoscale. The SiAlON wedge will be referred to as the Si$_3$N$_4$ sample for the remainder of this paper.

**Fig. 4:**
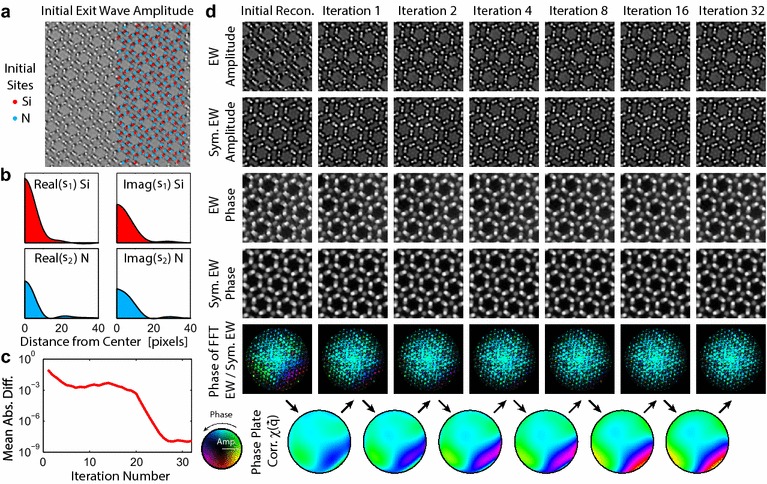
Residual aberration correction applied to a focal series reconstruction of a $\beta$-Si$_3$N$_4$ sample. **a** Reference region of the exit wave with atomic sites shown on half of image. **b** Complex atomic shape functions $\mathbf {s}_1$ and $\mathbf {s}_2$ for Si and N respectively. **c** Mean absolute difference of exit wave between each iteration. **d** Progression of the aberration correction algorithm, showing amplitude and phase of current corrected exit wave and symmetrized exit waves, phase difference of the Fourier transforms, and the fitted aberration function $\chi (q_x,q_y)$ with an outer radius of 15 nm$^{-1}$

Figure 4 shows the application of the above method for measuring residual aberrations to a focal series reconstruction of the Si$_3$N$_4$ sample. A reference region was selected near the middle of the reconstructed exit wave, the amplitude of which is shown in Fig. 4a. Two atom types are included (Si and N sites), and the same shape function is used for the two unique N sites. Figure 4c, d show that the aberration measurement is essentially converged after  5 iterations, and after 20 iterations the exit wave update approaches zero. The reconstruction algorithm therefore quickly evolves the aberration function coefficients towards the values which best approximate the point symmetrized exit wave. Note that the two are not exactly equivalent, as the experimental exit wave contains substantially more noise and can contain lattice distortions or small amounts of strain due to bending of the sample. No symmetrization is applied to the actual experimental exit wave in this approach. Furthermore, no additional filtering beyond the deconvolution with the residual wave aberration function and the informational limit cutoff have been applied to the experimental exit wave. The numerical aberration coefficients are given in the “Appendix”.

**Fig. 5:**
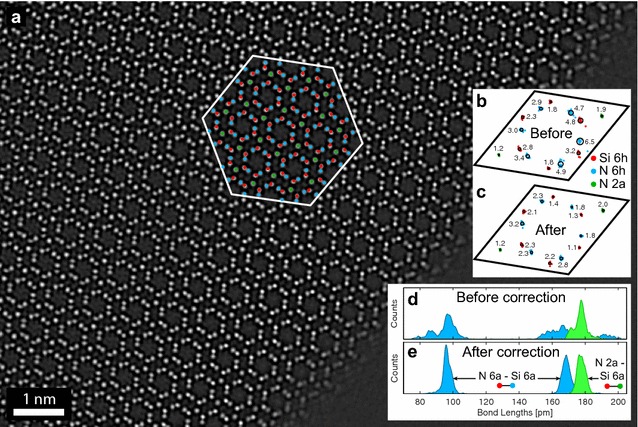
**a** Aberration-corrected exit wave amplitude of the Si$_3$N$_4$ sample. **b**, **c** Peak positions labeled in **a** plotted relative to positions inside the ideal unit cell for best-fit peaks before and after aberration correction respectively. All peak displacements from the ideal positions are scaled by a factor of 4 for the plotting, numbers indicate the RMS displacement in picometers. **d**, **e** Bond lengths from almost all peaks measured in image from before and after aberration-corrected images respectively

After measuring the residual aberrations from a small reference region, shown in Fig. 4, we have corrected these aberrations on the full image and plotted the amplitude in Fig. 5. The atomic positions appear extremely sharp, and no defects are visible other than the vacuum at the edge of the wedge sample. From multislice simulations we estimate the thickness of the crystalline portion of this sample ranges from 3 to 7 nm.

To quantify the atomic column positions, we used nonlinear least squares to fit the peak positions using a complex, two-dimensional elliptic Gaussian function. For the Si-N dumbbells, two complex elliptic Gaussian functions are fitted simultaneously. The fitted peak positions relative to the ideal Si$_3$N$_4$ lattice positions for a subset of 180 of the peaks are plotted in Fig. 5b, c from the exit waves before and after aberration correction. From the root-mean-square (RMS) displacements plotted, we see that the aberration correction has improved the fitting precision on most of the lattice sites. In particular, the dumbbells with strongly-overlapping peak functions have improved substantially, reaching peak precisions as low as 1.1 and 1.4 pm for the Si and N sites respectively. The isolated 2a N site position precision is not strongly affected by the residual aberrations.

Returning to the original question of measuring atomic shifts due to the doping, we have plotted the bond length distributions of all nearest-neighbor sites that are more than 2 unit cells distance from the vacuum edge and the edge of the full micrograph, in Fig. 5d, e. Before aberration correction, the bond length distribution for the dumbbell Si-N and the 2a N site –Si bonds appears to follow a bimodal distribution. The larger Si-N bond spacing in the hexagonal rings is even more distorted, spreading over approximately 50 pm. However after correcting the residual aberrations, all bond length distributions become monomodal. Therefore we found no evidence of systematic shifts in the 2a N sites. Additionally, no local distortions of the $\upbeta$-Si$_3$N$_4$ lattice such as those described in [39] were observed in this experiments. Finally, we note that because the reference lattice contains 14 site locations in each unit cell where measurements are taken, it is highly unlikely that we could be forcing one of the sites (such as a systematic 2a distortion) to be at an incorrect location. The algorithm should select the phase plate function which best minimizes the global aberrations.

### Single-layer graphene grain boundary

**Fig. 6:**
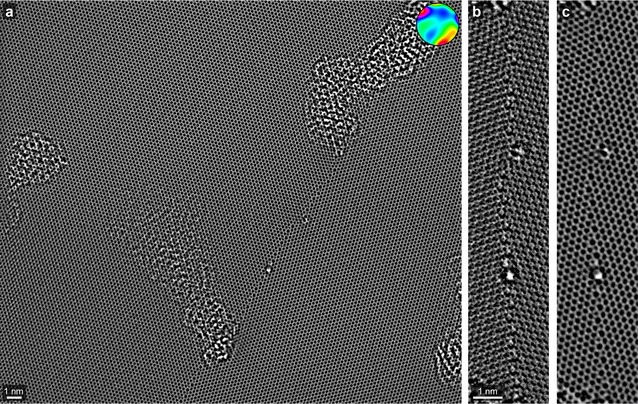
**a** Phase of an aberration-corrected exit wave of single-layer graphene, containing a grain boundary. Fitted aberration function is shown inset with an outer radius of 8 nm$^{-1}$. **b**, **c** Enlarged view of the unobstructed boundary before and after residual aberration correction respectively

The Si$_3$N$_4$ sample analyzed in the previous section did not contain any lattice defects, making it a relatively easy test of the correction algorithm described in this paper. A better test of our method would be a crystalline sample that contains a large number of possible local bond angles and lengths, allowing for many possible measurement errors due to residual aberrations. One such sample is the incommensurate grain boundaries found in polycrystalline single-layer graphene [32, 40, 41]. Figure 6a shows the exit wave phase of a graphene grain boundary with a large field of view after applying the aberration correction described in this work, with the aberration function inset and the numerical coefficients given in the “Appendix”. Topological variation in the sample has created regions where amorphous carbon can collect on the sample surface, but the majority of the field of view shows clean, defect-free graphene. Near the center of the field of view, an incommensurate boundary runs vertically.

The phase of the unobstructed region of the graphene grain boundary is plotted in Fig. 6b, c for the uncorrected and corrected exit waves respectively. Before aberration correction, we observe that the graphene lattices are extremely regular, but contain very little interpretable information. The grain boundary is particularly messy, due to the complex interaction of non-radially symmetric residual aberrations with the various atomic spacings present. By contrast, the corrected phase image in Fig. 6c has very well resolved atomic sites both in the crystalline lattices and along the grain boundary. Almost every site can be identified and the boundary structure can be easily quantified. We have used focal series exit wave reconstruction and the aberration correction algorithm described in this paper to characterize the structure of many different single-layer graphene grain boundary misorientations [42–44].

### Off-axis hologram of a silicon wedge

**Fig. 7:**
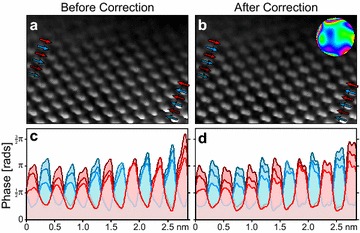
**a**, **b** Phase images of an off-axis hologram measurement of a Si sample with an [011] orientation, for the uncorrected and corrected exit waves respectively. Fitted aberration function is shown inset in **b** with an outer radius of 12 nm$^{-1}$. **c**, **d** Line traces taken from **a** and **b** respectively

The experimental exit waves in the previous two sections were reconstructed from focal series. Focal series reconstruction has a well-known limitation that it cannot accurately reconstruct lower spatial frequencies [12, 16]. This leads to exit wave phase images in the reconstructions that are somewhat flatter (lower peak-to-peak range) than the true exit wave phases. By contrast, since off-axis holography uses a reference wave created by an electron biprism, it can measure the absolute phase of an exit wave [17, 18]. In order to test our method on an exit wave containing the full range of spatial frequencies, we have recorded an off-axis hologram of a silicon wedge with an [011] orientation. The phase of this reconstructed exit wave is plotted in Fig. 7a. We have then applied our residual aberration correction algorithm to this measurement, shown in Fig. 7b. The numerical aberration coefficients are given in the “Appendix”.

The range of phases measured in these images is substantially higher than those in the previous focal series measurements, almost $2 \pi$ along the thinnest edge of the sample. After aberration correction, the Si dumbbells are more cleanly resolved. To show the dumbbell structure more clearly, we have plotted line traces in Fig. 7c, d, for the uncorrected and corrected phase images respectively. After correction, almost all dumbbells show clear separation between the two Si atomic columns.

## Conclusion

We have developed an algorithm for measuring and correcting residual coherent wave aberrations in complex exit waves of crystalline samples, measured in transmission electron microscopy. Our algorithm relies on creating a synthetic exit wave by applying point-symmetrization to all atomic columns in a reference region, to approximate the aberration-free exit wave. Because our method is objective and automatic, it is not prone to operator errors that could be introduced from manual correction of the residual aberrations. It is important to note that no symmetrization is applied to the final experimental exit wave. We have applied our method to three experimental datasets, focal series reconstructions of a Si$_3$N$_4$ wedge and a single-layer graphene grain boundary, and an off-axis hologram of a silicon wedge. In all cases, the residual aberration correction improved the precision, accuracy and interpretability of the complex exit waves. Our algorithm is simple to implement, and applicable to a large class of experimental exit wave measurements of crystalline samples oriented along a low-index zone axis.

## Appendix

The numerical aberration coefficients found in this study are given in Table 1, using the convention used by CEOS aberration correctors [7]. In all images, the origin is in the upper left corner and the x-axis points down while the y-axis points right.

**Table 1:** Numerical aberration coefficients measured in this study for the Si$_3$N$_4$, graphene grain boundary and silicon wedge experimental datasets

Order		CEOS	Si$_3$N$_4$		Graphene		Si Wedge	
m	n	Name	$C^x$	$C^y$	$C^x$	$C^y$	$C^x$	$C^y$
0	2	$A_1$	0.7	2.0	4.0	−4.7	0.0	2.0 nm
0	3	$A_2$	−5.2	66.5	60.1	−112	−15.3	−35.7 nm
1	1	$B_2$	10.4	67.6	−123	−79.8	23.0	84.8 nm
0	4	$A_3$	0.6	0.2	−5.6	−2.8	−1.4	−1.6 μm
1	2	$S_3$	−1.5	0.0	−3.4	4.3	1.0	−1.4 μm
0	5	$A_4$	0.7	−4.6	−26.3	31.2	−7.9	5.6 μm
1	3	$D_4$	−6.7	−15.0	−26.1	18.7	8.6	12.6 μm
2	1	$B_4$	31.7	−47.8	99.2	55.7	−5.0	−30.3 μm
0	6	$A_5$	−1.4	0.3	−0.8	−3.1	0.0	0.0 mm
1	4	$R_5$	−0.2	-0.3	1.1	0.6	0.0	0.2 mm
2	2	$S_5$	2.6	−0.2	0.5	−1.9	−0.6	0.5 mm
